# Affectivity in danish patients with emotional disorders: assessing the validity of the Positive and Negative Affect Schedule (PANAS)

**DOI:** 10.1186/s12888-023-05450-z

**Published:** 2023-12-13

**Authors:** Oliver Rumle Hovmand, Nina Reinholt, Anne Bryde Christensen, Anita Eskildsen, Bo Bach, Mikkel Arendt, Stig Poulsen, Morten Hvenegaard, Sidse M. Arnfred

**Affiliations:** 1 Psychiatric Research Unit, Region Zealand Mental Health Service, Faelledvej 6, 4200 Slagelse, Denmark; 2 Department of Clinical Medicine, Faculty of Health, University of Copenhagen, Copenhagen, Denmark; 3 Psychiatric Research Unit, Region Zealand Mental Health Services, Copenhagen, Denmark; 4 Psychiatry South, Region Zealand Mental Health Services, Copenhagen, Denmark; 5 Eating Disorders Research Unit, Mental Health Services, Copenhagen, Denmark; 6 Department of Affective Disorders, Aarhus University Hospital, Aarhus, Denmark; 7 Department of Psychology, University of Copenhagen, Copenhagen, Denmark; 8 Neurocentre, Rigshospitalet, Copenhagen University Hospital, Copenhagen, Denmark

**Keywords:** Affectivity, Emotional disorders, PANAS

## Abstract

**Background:**

The Positive and Negative Affect Schedule (PANAS) was designed to measure trait positive affect (PA) and trait negative affect (NA).

**Methods:**

The Danish PANAS was administered to outpatients with depression and anxiety disorders. Internal consistency was assessed using Cronbach’s alpha and McDonald’s omega and factorial structure was evaluated using confirmatory factor analysis (CFA). Convergent validity was evaluated by means of correlations with the negative affectivity and the detachment domain of the Personality Inventory for DSM-5 Short Form (PID-5-SF), the Hamilton Anxiety Rating Scale 6 (HARS-6) and the Hamilton Depression Rating Scale 6 (HDRS-6).

**Results:**

PANAS Scores of 256 patients were analyzed. Cronbach’s alpha and McDonald’s omega showed good internal consistency for both the PA score (alpha = .84 and omega = .89) and the NA score (alpha = .86 and omega = .90). CFA analysis confirmed a structure with two factors corresponding to the PA and NA factors. PA was negatively correlated with the detachment domain of PID-5 (*r* = -.47), HARS-6 (*r* = -.15) and HDRS-6 (*r* = -.37). NA was positively correlated with PID-5-SF negative affectivity domain (*r* = .43), HARS-6 (*r* = .51) and HDRS-6 (*r* = .52).

**Discussion:**

The Danish PANAS has promising internal consistency and construct validity, which are comparable to other studies of the instrument.

**Supplementary Information:**

The online version contains supplementary material available at 10.1186/s12888-023-05450-z.

## Background

The Positive and Negative Affect Schedule (PANAS) was developed by Watson (1988) and is designed to assess two distinct temperamental or personality traits, described as dimensions of affectivity [1]. Their distinction is based on the theoretical work by Bradburn (1969) [2], who describes positive affect (PA) and negative affect (NA) as two relatively dominant and distinct dimensions of emotion. PA assesses the “extent to which respondents feel enthusiastic, active, and alert [1], and NA a variety of aversive mood states, such as anger, anxiety, fear, and guilt. NA is conceptualized similarly to the Big Five personality trait of neuroticism. In NEO Personality Inventory, Costa and McCrae (1992) conceptualize neuroticism as a general tendency to experience negative emotions such as embarrassment, fear, sadness, anger, disgust, and guilt. Further, the Personality Inventory for DSM-5 Short Form (PID-5-SF) has been developed to assess the more pathological aspects of the same personality dimensions (e.g., emotional lability, anxiousness, separation insecurity) [3, 4].

Instruments measuring NA and PA are strongly correlated with measures of anxiety disorders and depression [5–8] and the development and maintenance of unipolar depression, anxiety, and other emotional disorders have been proposed to be intricately linked to NA [9–11]. Likewise, low levels of PA are associated with symptoms of depression [12] and anxiety [1] and can predict the onset of depression [12], while high levels of PA are associated with greater psychological well-being [13] and a state of calmness and serenity [1].

Watson et al. (1988) [1] utilized the work of Zevon and Tellegen (1982) [14] when developing the PANAS. They identified 60 different words used to describe mood and constructed 20 different content categories (e.g., attentive, distressed, excited) to which the words could be assigned. From those 60 words, Watson et al. (1988) selected the one, which exhibited high loadings on one factor (PA or NA) and loaded little to none on the other factor. Subsequently, the factorial validity was examined for six different timeframes (e.g., moment, past week) in samples primarily consisting of university students. In accordance with the theoretical structure, principal factor analyses found that the items loaded onto two dominant factors.

Since the introduction of the PANAS, several studies have explored the factor structure of the PANAS through exploratory or confirmatory factor analysis (CFA). The PANAS was originally hypothesized as having two independent factors, but CFA has generally failed to find support for this factor structure [15–18]. A model where the two factors are allowed to correlate has been proposed, and studies have generally found a better fit to this factor structure [19, 20]. This model also exist in a variation proposed by Mackinnon et al. (1999) [21] where the item “excited” is allowed to load onto both PA and NA. Finally, a two factor model proposed by Crocker (1997) [22] where the two factors are allowed to correlate, and where residuals also correlates if the items belonged to the same content category (e.g., Exited and Enthusiastic correlates because they belong to the same content category) set forth by Zevon and Tellegen (1982) have been proposed [14]. A meta-analysis by Wedderhoff et al. (2021), which analyzed data from 57 independent samples (*N* = 54,043), found support for this modified two-factor model [23].

The original English PANAS has been validated in several non-clinical samples across different cultures and languages (e.g., Estonian, Hungarian, German, Korean, Japanese, Portuguese, Romanian, Spanish, Russian and Turkish), and the results of these validation studies have consistently shown satisfying psychometric properties of the scale [15, 16, 19, 24–29].

In this study, we report data on the internal and construct validity of the Danish PANAS in a sample of patients with emotional disorders awaiting cognitive behavioural therapy in outpatient psychiatric clinics.

### Aims and hypotheses of the study

The aim of the present study was twofold: (a) to evaluate the internal consistency and confirm the factorial structure of the Danish PANAS in a clinical sample; (b) to examine the construct validity of the PANAS by means of associations with trait dimensions of detachment and negative affectivity as well as clinician rated measures of anxiety and depression.

We hypothesized that the Danish PANAS is structurally composed of two distinct factors of affect corresponding to NA and PA. We further hypothesized a positive correlation between the NA factor and anxiety, depression, and the PID-5-SF negative affectivity domain and a negative correlation between the PA factor and measures of anxiety, depression, and the PID-5-SF detachment domain.

## Methods

### Data sample

Data for the present study were collected as part of a multi-center randomized controlled non-inferiority trial comparing diagnosis-specific cognitive behavioral therapy (CBT) to transdiagnostic group CBT in Danish outpatient psychiatric clinics [30]. These clinics provide time-restricted, standardized treatment programs for emotional disorders to patients referred by their general practitioner or private practice psychiatrist, if they have not responded to treatment in the primary healthcare system, have substantial impairment of functioning, or a substantial worsening of symptoms. The included patients were diagnosed with the Mini International Neuropsychiatric Interview (MINI) [31] in addition to the routine clinical diagnostic assessment administered in the given clinics. Data for the present study were collected at baseline while the included patients awaited therapy (see [32] and [30] for further details of the mother study).

### Translation

NR ensured permission from David Watson to translate the PANAS into Danish for research purposes. The PANAS was first translated into Danish by an expert panel, and this version was then translated back into English. The back-translation was presented to David Watson, who approved it. The result of this translation is presented in Additional File 1.

### Instruments

#### The Positive and Negative Affect Schedule (PANAS)

The PANAS is a 20-item self-report schedule designed to provide brief measures of both PA and NA [1]. The included items were derived from a principal component analysis of Zevon and Tellegen’s mood checklist [14]. Respondents are asked to rate the degree to which they have experienced a number of particular emotions (e.g., alert, inspired, enthusiastic) within the last week. The items are scored on a five-point Likert scale (1 = Very slightly or not at all, 2 = Little, 3 = Moderately, 4 = Quite a bit, 5 = Very much).

#### Hamilton Anxiety Rating Scale 6 (HARS-6)

HARS-6 is a shortened version of the original 14-item Hamilton Anxiety Scale (HAM-A) [33]. It is clinician-administered and covers six aspects of anxiety disorders as one homogenous factor (total score). Items are scored on a five-point Likert-scale (0 = Not present, 1 = Mild, 2 = Moderate, 3 = Severe, 4 = Very severe). The scale has shown high discriminant validity [34, 35]. In the present study, the HARS-6 was based on a telephone interview made by trained research assistants.

#### Hamilton Depression Rating Scale 6 (HDRS-6)

HDRS-6 is a shortened version of the original 17-item Hamilton Depression Rating Scale (HAM-D) [36]. It is clinician-administered and covers six aspects of depressive disorders as one homogenous factor (total score). Items are scored on a five-point Likert-scale (0 = Not present, 1 = Mild, 2 = Moderate, 3 = Severe, 4 = Very severe). It has been shown to reflect the total HAM-17 score [37, 38]. HDRS-D6 was administered based on a telephone interview made by trained research assistants.

#### Personality Inventory for DSM-5 Short Form (PID-5-SF)

PID-5-SF is an abbreviated 100-item version of the original 220-item Personality Inventory for DSM-5 (PID-5), developed to measure the pathological trait specifiers listed in the alternative model for personality disorders (AMPD) in DSM-5 Section III [3, 4]. Participants were required to rate each PID-5-SF item on a 4-point scale (0 = Very false” or Often false to 3 = Often true or Very true). In the present study, we only employed the domain scales of Negative Affectivity and Detachment. The Danish translation of the PID-5-SF has shown sound psychometric qualities [39, 40].

### Statistical procedures

We undertook all data processing and analyses using R 4.3.0 (Already Tomorrow) and RStudio 2022.07.2 + 576 [41], including the psych 2.1.9 [42] and Lavaan 0.6–9 [43] R packages. R scripts are available from the first author upon request. Demographic information is reported as frequencies and percent; PANAS scores as means, standard deviations and range. The PANAS employs Likert-type responses with 5 points and non-normality in the distribution of responses. All data was therefore treated as ordinal and analyzed as categorial.

First, we assessed the internal validity and reliability of the Danish PANAS by calculating the Cronbach’s alpha (*α*), the MacDonald’s omega hierarchical (*ω*_h_) and omega total (*ω*_total_) for the two expected factors (PA and NA). Alpha and omega total above 0.70 are considered satisfactory [44, 45]. We determined the internal consistency using McDonald’s ω rather than Cronbach’s α because it is favored as a more optimal and robust measure of scale reliability, particularly when unidimensional latent constructs can be estimated [46].

Secondly, we carried out confirmatory factor analyses (CFA) of the Danish PANAS to calculate its fit with four different models suggested by previous factor analyses [16, 23]. As recommended by Kline (2015), [47] we calculated the comparative fit index (CFI), the Tucker-Lewis index (TLI), the root mean square error of approximation (RMSEA), the standardized root mean square residual (SRMR), and the degrees of freedom (df). The consensus is that a larger RMSEA and smaller CFI and TLI values indicate a worse fit [48]. More specifically, we utilized the criteria set forth by Hu and Bentler, which suggests that an RMSEA smaller than 0.06, an SRMR smaller than 0.08, and a CFI and TLI larger than 0.95 indicate relatively good model–data fit [49]. The chi-square fit statistic is affected by large samples and is therefore usually evaluated as the ratio of the chi-square statistic to the respective degrees of freedom (χ2 /df) [50]. A ratio of smaller than 2 indicates a superior fit data [51]. We also drew a figure with factor loadings of the model, which had the best fit of the ones examined.

Lastly, we evaluated the construct validity of the individual factors of the Danish PANAS by calculating Pearson’s correlations with the Negative Affectability and Detachment domains of the PID5-SF and HARS-6, the HDRS-6 [39]. Correlations less than 0.30 were considered negligible, correlations between 0.30 and 0.50 were considered low, correlations between 0.50 and 0.70 were considered moderate, and correlations above 0.70 were considered high [52].

## Results

Two hundred and ninety-one patients were included in the original sample. There were no data on the PANAS from 35 patients, who were removed from the sample. This left a sample of 256 patients, whose data were analyzed in the present study. See Table 1 for characteristics of the included patients and Table 2 for PANAS scores of the different diagnostic categories. The sample was predominantly young and female, and almost half were well-educated. Most were diagnosed with unipolar depression (48.4%) or social anxiety disorder (28.5%). Further, 56.64% of the included patients had any secondary diagnosis and 24.6% also had a tertiary diagnosis. The most frequently observed secondary diagnoses were anxiety and depression disorders. Patients with any psychotic illness, any known personality disorder, and high risk of suicidality were excluded from the trial. Most of the included patients showed a long-term course of illness with a mean duration of 4.7 years. Moreover, 41.4% of the sample were currently on long-term sick leave. See Table 3 for summary statistics of the Danish PANAS.

**Table 1:** Sample characteristics regarding demographic information, diagnosis, age and duration of illness

	Total (*n* = 256)
Age, years	32.32 (11.01)
Duration of current episode, years	4.72 (8.04)
Female	143 (63.28%)
Married/in a partnership	126 (49.21%)
Professional or university graduate	119 (46.48%)
Employment	
Full time/part time/student	95 (37.11%)
Sick leave > 3 months	106 (41.40%)
Retired or unemployed	24 (9.37%)
Other	31 (12.11%)
Principle diagnosis	
Depression	124 (48.44%)
Social Anxiety Disorder	73 (28.51%)
Panic disorder	45 (17.58%)
Agoraphobia	14 (5.47%)

Data are presented as n (%) or mean (SD)

**Table 2:** Summary statistics of the Danish PANAS for the patients with depression, anxiety and panic disorder/agoraphobia separately

	**Median**	**Mean (SD)**	**Range**
Depression (*n* = 124)			
PA	18	19.35 (5.43)	10–39
NA	28	28.08 (7.62)	10–45
Social Anxiety Disorder (*n* = 73)			
PA	20	21.37 (5.72)	11–40
NA	26	27.25 (8.83)	12–48
Panic disorder (*n* = 45)			
PA	23	23.71 (6.73)	12–44
NA	29	29.6 (8.24)	14–46
Agoraphobia (*n* = 14)			
PA	23	22.29 (6.99)	11–35
NA	32	31.71 (4.79)	22–39

**Table 3:** Summary statistics of the Danish PANAS for the total sample and males and females separately

	**Median**	**Mean (SD)**	**Range**
Total sample (*n* = 256)			
PA	20	20.85 (6.04)	10–44
NA	29	28.31 (8.01)	10–48
Males (*n* = 94)			
PA	19	20.05 (6.33)	11–40
NA	28	27.96 (7.82)	12–43
Females (*n* = 162)			
PA	21	21.31 (5.84)	10–44
NA	29	28.51 (8.13)	10–48

### Reliability of the Danish PANAS

Internal consistencies were found to be satisfactory for the individual factors (Positive affect *α* = 0.84, 95% CI [0.81, 0.87] *ω*_h_ = 0.69, *ω*_total_ = 0.89; Negative affect; *α* = 0.86, 95% CI [0.83, 0.89], *ω*h = 0.65, *ω*_total_ = 0.90).

### Factor structure of the Danish PANAS

Table 4 presents the fit statistics for the factor structures using confirmatory factor analysis. Overall, none of the models had a good fit to data. However, the modified two-factor model allowing the factors and residuals to correlate had superior fit to data on the CFI, the TLI, the Chi2/Df index, and the PCF. However, the two-factor model where factors were allowed to covariate had the best fit to data on the SRMR and the RMSEA.

**Table 4:** Fit statistics for four different possible factor structures of the Danish PANAS

Model	CFI	TLI	RMSEA	SRMR	Chi2 (*p*-value)	Df	Chi2/Df	PCF
Single-factor model	.496	.436	.150 [.142, .159]	.164	1152.743 (.000)	170	6.78	.539
Two-factor model	.795	.769	.096 [.088, .105]	.107	568.873 (.000)	169	3.36	.808
Two-factor model, excited cross-loads	.769	.832	.096 [.087, .105]	.107	565.416 (.000)	168	3.36	.647
Two-factor model, factors allowed to correlate	.795	.769	.096 [.088, .105]	.096	568.873 (.000)	190	2.99	.650
Two-factor model, factors and residuals correlate	.877	.850	.078 [.068, .087]	.100	395.937 (.000)	156	2.54	.869

*N* 256, *CFI* comparative fit index, *TLI* Tucker-Lewis index, *RMSEA* root mean square error of approximation, *SRMR* standardized root mean square residual, *Df* degrees of freedom, *Chi2* Chi Square, *PCF* Probability of Close Fit

**Fig. 1:**
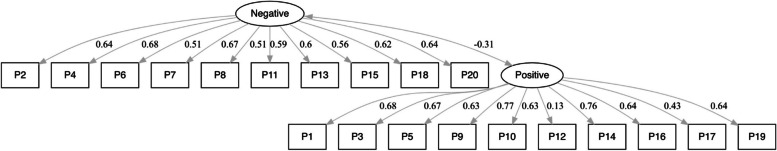
Graphical representation of the factor structure of the two-factor model of the Danish PANAS. Negative = Negative affect, Positive = Positive affect, PX = Item number X of the Danish PANAS. Ovales represents latent variables / factors. Squares represents individual items

Figure 1 shows the factor structure and factor loadings for the modified two-factor model allowing the two factors and residuals to correlate.

### Convergent validity

The Pearson’s correlations between the Danish PANAS and measures of anxiety, depression and maladaptive personality traits are presented in Table 5. We found a low negative correlation between the PA factor and the Detachment Domain of the PID-5-SF, and a negligible correlation with symptoms of anxiety and depression. Likewise, the NA factor showed a low positive correlation with the PID-5-SF Negative Affect domain and a moderate correlation with symptoms of anxiety and depression. The correlations were markedly stronger for NA than for PA. PA correlated negatively, but negligibly with NA.

**Table 5:** Pearson’s Correlations among PA, NA, and measures of personality traits, depression and anxiety

	**Measure**	**Correlation**	*P*
PA	PID-5-SF Detachment	-.47 95% CI [-.56, -.37]	> .01
PA	HARS-6	-.15 95% CI [-.27, -.02]	.02
PA	HDRS-6	-.37 95% CI [-.47, -.26]	> .01
PA	NA	-.16 95% CI [-.27, -.04]	.01
NA	PID-5-SF Negative Affect	.43 95% CI [.32, .53]	> .01
NA	HARS-6	.51 95% CI [.42, .60]	> .01
NA	HDRS-6	.52 95% CI [.42, .60]	> .01

*PA* Positive affect, *NA* Negative Affect, *HARS-6* Hamilton Anxiety Rating Scale 6, *HDRS-6* Hamilton Depression Rating Scale 6, *PID-5-SF* Personality Inventory for DSM-5 Short Form (PID-5-SF)

## Discussion

The Danish PANAS showed good internal consistency, which is in accordance with findings in a validation study on the Korean PANAS in a sample of psychiatric outpatients with emotional disorders (alpha of 0.91 for the NA factor and 0.87 for the PA factor) [25]. Other studies of PANAS in psychiatric outpatients have not, to our knowledge, published measures of internal consistency [53, 54]. Further, we are not aware of any studies reporting omega values, and we can therefore not judge how our findings compare to previous research. Our results regarding internal consistency are also in line with investigations of the British PANAS in healthy volunteers (alpha of 0.85 for the NA factor and 0.89 for the PA factor) [16] and the Portuguese PANAS in an elderly, healthy population (alpha of 0.88 for the NA factor and 0.92 for the PA factor) [55]. We provide summary statistics of the Danish PANAS for individuals with emotional disorders. Interestingly, we found that the patients suffering from agoraphobia rated more pronounced negative affect than the other diagnoses.

We further evaluated the factor structure of the Danish PANAS. The two-factor model where the two factors were allowed to correlate had the best fit to data on most fit indices. This two-factor model was supported by confirmatory factor analysis. Hence, our results are in line with the findings of a meta-analysis of the factor structure of the PANAS by Wedderhoff et al. (2021), who evaluated 57 independent samples (N = 54,043) of data on both the original PANAS and translations of the instrument, and who also found best support for this model [23]. The fit was not perfect, but it was the best fit among the evaluated models. These findings support the hypothesis that the PANAS indexes two distinct, but also moderately negatively correlated factors of affect. But the finding that NA and PA are negatively correlated does, as previous findings who have showed the same [16], seem to challenge the original assumption that NA and PA are independent dimensions.

We generally found smaller loadings of the individual items in the Danish PANAS, compared to the loadings in the original version presented by Wedderhoff et al. (2021) [23]. Especially on the PA, item loadings were markedly smaller in the Danish PANAS compared to the English PANAS. This finding could be explained by either the procedure for which the PANAS was translated into Danish, or subsequently the quality of the translation. Since the instrument was translated according to relevant guidelines, this might not be the best explanation of the variance. One possible explanation is that items of the PANAS could be interpreted differently in different cultures. It might therefore be the case that some items of the PANAS reflect an American or English understanding of positive emotions or negative emotions, which is not the same in the Danish culture.

We assessed the convergent validity of the Danish PANAS by investigating its correlation with measures of anxiety, depression, Negative Affectivity, and Detachment. We found a low positive correlation between PID-5 Negative Affectivity and the NA factor of the Danish PANAS and a low negative correlation between the PA factor and Detachment domains. This suggests that the instruments measure related but different concepts. This finding is expected, since both the PID-5 and the NA of the PANAS ask about negatively charged emotions. However, the aim of the PANAS is to quantify recent intensity and surveyed patients are therefore asked to rate the severity of specific symptoms within the time frame of a week, while the PID-5 Negative Affectivity aims to quantify personality traits, and therefore asks patients if they agree with more general, global descriptions of tendencies about themselves. Hence, the timeframe covered by our translation of the PANAS covers trait NA, while the PID-5 covers state NA. It was also expected that the PANAS PA was negatively correlated with the Detachment domains, since the Detachment domain is designed to capture the opposite of PA (e.g., low extraversion, depressive affect, and interpersonal). Again, one could have expected that the PA and the Detachment domain would have correlated negatively to a larger degree. However, as it was the case with the NA and the PID-5 Negative Affectivity, this could possibly also be explained by the fact that NA assesses trait and PID-5 state negative affectivity.

We are unaware of other studies that have reported the association between PID-5 Negative Affectivity and Detachment and PANAS. Accordingly, we are unable to compare our findings to other studies.

Further, we demonstrated that the NA factor was positively correlated with symptoms of both anxiety and depression, but that the PA factor was more negatively correlated with symptoms of depression than with those of anxiety (-0.37 vs -0.15). These results are in line with findings in studies of American outpatients [54], as well as the study of the Korean PANAS which found similar correlations with measures of depression. However, in contrast to findings presented here, the Korean study only found that the PA factor was associated with symptoms of depression and not with anxiety [25]. Our results are also aligned with studies on the British PANAS in healthy volunteers, where the PA factor was negatively correlated with symptoms of depression but less so with symptoms of anxiety [16]. An explanation of this could be that symptoms of anxiety are relatively common in healthy individuals but that the anxiety experienced in this population was not strong enough to be accompanied by depressive symptoms. The correlations with PID-5 SF domains were unexpectedly less pronounced than the correlations with symptoms of anxiety and depression.

### Limitations

A few limitations of this study must be noted, when interpreting the results. First, our dataset only included data from 256 patients and the validity of our findings might be increased with a larger sample. Second, the validation was conducted in a relatively homogenous sample of patients with moderate to severe emotional disorders [30]. Therefore, generalizability of our findings to different clinical and non-clinical samples needs to be established. Lastly, our study did only include one test of measurement invariance and did not investigate and compare intercept and residuals for the Danish PANAS with data for the original PANAS.

These limitations could be addressed in future research on the Danish PANAS, and such research could be conducted in the background population and various other patient samples suffering from different psychiatric disorders. This could strengthen the understanding of the instrument’s usefulness in Danish-speaking populations. Future studies of measurement invariance could also provide data on how comparable the Danish PANAS is to the original. Normative data for the general population could be a further improvement.

In conclusion, our findings indicate that the Danish PANAS has the same fair to good psychometric properties as the original English version and is best described by a factor structure corresponding to the one supported by a meta-analysis of studies on the original English version. In support of our study hypotheses, we found that the NA factor is positively correlated with another type of questionnaire assessment of NA (PID-5 Negative Affectivity) and symptoms of anxiety and depression, and that the PA factor is negatively correlated with the PID-5 trait of Detachment, conceptualized as the reciprocal of Positive Affectivity, and symptoms of anxiety and depression. These findings help to establish convergent validity of the Danish PANAS.

In conclusion, the current study assessed the psychometric properties of the Danish PANAS in an outpatient population, and the results indicate that it is a valid and sensitive measure of positive and negative affect, which is comparable to the English version.

### Supplementary Information

**Additional file 1.**

## Data Availability

By reasonable request from the last author.
