# Supplementary material for: Affectivity in danish patients with emotional disorders: assessing the validity of the Positive and Negative Affect Schedule (PANAS)
Source: BMC Psychiatry. 2023 Dec 13;23:943. doi: 10.1186/s12888-023-05450-z (PMC10720164; doi:10.1186/s12888-023-05450-z)
Supplement: Supplementary file 1 — Additional file 1. [file 12888_2023_5450_MOESM1_ESM.docx]

**The positive and negative affect scale (PANAS)**

The Danish translation is presented on page 2. The original English edition is presented on page 3.

Dette skema består af en række ord der beskriver forskellige følelser og tilstande. Læs hvert ord grundigt og angiv ved hjælp af nedenstående skala et tal for hvert ord. Angiv ved hjælp af skalaen, i hvilken grad du har følt på denne måde *i den forløbne uge*.

| 1 | 2 | 3 | 4 | 5 |
| --- | --- | --- | --- | --- |
| Meget lidt eller slet ikke | Lidt | Moderat | En hel del | Ekstremt meget |

| 1 | Interesseret | _____________ | 11 | Irritabel | _____________ |
| --- | --- | --- | --- | --- | --- |
| 2 | Forpint | _____________ | 12 | Årvågen, beredt | _____________ |
| 3 | Begejstret | _____________ | 13 | Skamfuld | _____________ |
| 4 | Oprevet | _____________ | 14 | Inspireret | _____________ |
| 5 | Stærk | _____________ | 15 | Nervøs | _____________ |
| 6 | Skyldig | _____________ | 16 | Beslutsom | _____________ |
| 7 | Skræmt | ____________ | 17 | Opmærksom | _____________ |
| 8 | Fjendtlig | _____________ | 18 | Rastløs, urolig | _____________ |
| 9  10 | Entusiastisk  Stolt | _____________  _____________ | 19  20 | Aktiv  Bange | _____________  _____________ |

This scale consists of a number of words and phrases that describe different feelings and emotions. Read each item and then mark the appropriate answer in the space next to that word. Indicate to what extent you have felt this way during the past few weeks.

| 1 | 2 | 3 | 4 | 5 |
| --- | --- | --- | --- | --- |
| Very slightly or not at all | A little | Moderate | Quite a bit | Extremely |

| 1 | Interested | _____________ | 11 | Irritable | _____________ |
| --- | --- | --- | --- | --- | --- |
| 2 | Distressed | _____________ | 12 | Alert | _____________ |
| 3 | Exited | _____________ | 13 | Ashamed | _____________ |
| 4 | Upset | _____________ | 14 | Inspired | _____________ |
| 5 | Strong | _____________ | 15 | Nervous | _____________ |
| 6 | Guilty | _____________ | 16 | Determined | _____________ |
| 7 | Scared | ____________ | 17 | Attentive | _____________ |
| 8 | Hostile | _____________ | 18 | Jittery | _____________ |
| 9  10 | Enthusiastic  Proud | _____________  _____________ | 19  20 | Active  Afraid | _____________  _____________ |
